# Successful Surgical Correction of Astigmatism using Customized Ablation Photorefractive Keratectomy

**Published:** 2016

**Authors:** Hakimeh TAHERI, Shahrokh RAMIN

**Affiliations:** 1 Ophthalmic Research Center, Shahid Beheshti University of Medical Sciences, Tehran 14416, Iran

**Keywords:** Photorefractive Keratectomy, Customized Ablation, Excimer Laser, Astigmatism

## Abstract

The aim of this study was to determine the change in the degree of astigmatism in patients treated with customized ablation photorefractive keratectomy (PRK). This is a cross-sectional study that involved 92 otherwise healthy subjects with regular and irregular astigmatism ≥ 1.25 D (mean age: 39.09 ± 7.72 years; range: 20–59 years). All study subjects were treated with customized ablation PRK using a Technolas 217p Excimer Laser System. Before and 6 months after the surgery, a refraction assessment was conducted for each subject, and the effectiveness of the surgery for correcting astigmatism was evaluated. There was a significant change in astigmatism based on the results of an automated refraction exam of -1.67 ± 1.03 D (P < 0.001), from -2.51 ± 1.45 D preoperatively to -0.87 ± 0.94 D postoperatively. There was also a significant change in subjective refraction of -2.00 ± 1.25 D (P < 0.001), from -2.46 ± 1.52 D preoperatively to -0.46 ± 0.97 D postoperatively. Therefore, our results show that customized ablation PRK is effective for correcting astigmatism ≥ 1.25 D (P < 0.001).

## Introduction

Astigmatism accounts for 13% of refractive errors, of which myopia comprises 35.4% and hyperopia, 5.04%. Further, it is quite a challenging disorder to manage for both the patient and the treating ophthalmologist (1, 2). An optical aberration is a deviation from the ideal position of the light after transmission through the optical system(based on simple paraxial theory) that affects the quality of an individual’s vision (3). Stigmatic image formation by both the human eye and other optical systems is problematic due to the existence of optical aberrations such as astigmatism. Therefore, improvement of higher-order aberrations can improve visual acuity in humans (4). However, this type of aberration cannot be improved using ordinary refractive corrections (such as those involving lenses with sphere or cylinder power), although these treatments can be used for the correction of lower-order aberrations (5).

Higher-order aberrations such as astigmatism can lead to the development of an aura, field curvature, impaired contrast sensitivity, and impaired night vision (6, 7). Even after successful refractive surgery, this type of the aberration reoccurs in 30% of patients (8, 9), thereby significantly impairing vision (10). Customized ablation is essential to improve visual acuity by correcting the refractive error and avoiding optical aberrations (11). Wavefront-guided photorefractive keratectomy (PRK) has been shown to be capable of optimizing these visual factors (12). In addition to refractive correction and the correction of pre-existing higher-order aberrations, PRK can simultaneously prevent surgically induced aberrations, and studies in normal subjects have shown that optical quality improves after PRK (13, 14). Customized ablation PRK can prevent higher-order aberrations, and hence, it could be a useful surgical treatment for astigmatism. We conducted this study to evaluate the effectiveness of customized ablation PRK in patients with astigmatism ≥ 1.25 D.

## MATEIALS AND METHODS

In this cross-sectional study, customized ablation PRK was performed on 92 otherwise healthy subjects (44 men and 48 women) with preoperative astigmatism ≥ 1.25 D from January 2012 to June 2015 at Negah Eye Hospital, Tehran, Iran. The study was approved at the department of optometry, Shahid Beheshti University, and informed consent was obtained from the subjects. The inclusion criteria consisted of regular or irregular astigmatic refractive error ≥ 1.25 D in otherwise healthy patients aged ≥20 years and stable refraction ≥1 year prior to surgery. The exclusion criteria consisted of previous eye surgery, comorbid ocular conditions such as keratoconus (based on the Rabinowitz–McDonnell indices), family history of keratoconus, pregnancy, and breastfeeding. The preoperative examinations comprised an uncorrected distance visual acuity (UDVA) and a corrected distance visual acuity (CDVA) assessment (using a Snellen chart), retinoscopy, a refraction assessment using an autorefractor, and a complete eye examination (which included a subjective refraction assessment). The patients underwent customized ablation PRK with a Technolas 217p Excimer Laser System (Bausch & Lomb Inc., Rochester, NY). At 6 months after the surgery, an objective and subjective assessment of the surgical success rate were conducted. This success rate of the surgery for correcting astigmatism was determined by comparing the degree of pre- and postoperative astigmatism.

The data are presented as means, standard deviations, medians, ranges, and percentages. To compare the changes in astigmatism, we used t-tests and Mann–Whitney U tests. To compare the success rate of the surgery, we used chi-square test or Fisher’s exact test. Lastly, to compare the changes in each group, we used paired t-tests or Wilcoxon signed-rank test. Statistical analysis was conducted using SPSS (version 22.0, IBM Corp., Armonk, NY, USA), and p values < 0.05 were considered statistically significant.

## RESULTS

The patients were aged 20–59 years (mean age: 39.09 ± 7.72 years), as shown in Table 1, which also shows the other demographic characteristics, the preoperative UDVA and CDVA, and the postoperative CDVA of the subjects.

Table 2 shows the postoperative change in objective and subjective refractive error. According to the results of an automated refraction exam, there was a significant change in astigmatism of -1.67 ± 1.03 D (P < 0.001), i.e., from -2.51 ± 1.45 D preoperatively to -0.87 ± 0.94 D postoperatively. There was also a significant change in subjective refraction of -2.00 ± 1.25 D (P < 0.001), i.e., from -2.46 ± 1.52 D preoperatively to -0.46 ± 0.97 D postoperatively.

The results showed that customized ablation PRK was effective for correcting astigmatism > 1.25 D as there was a significant association between customized ablation PRK and reductions in astigmatism (P < 0.001), both in terms of objective refraction for 14 eyes (15.2%, 95% CI: 7.7–22.7%) and subjective refraction for 60 eyes (65.2%, 95% CI: 55.3–75.1%); no cylindrical component was noted postoperatively.

**Table 1:** Demographic characteristics and visual acuity of the subjects

**Variable**	**Values**
**Age**	
**Mean±SD**	33.09 ± 7.72
**Median (range)**	33(20– 59)
**Sex**	
**Men Frequency (%)**	44 (47.8%)
**Women Frequency (%)**	48(52.2%)
**Preoperative CDVA**	
**Mean±SD**	0.1 ± 0.42
**Median (range)**	0(0– 2.3)
**Preoperative UDVA**	
**Mean±SD**	0.16 ± 0.35
**Median (range)**	0(0– 1.61)
**Postoperative CDVA**	
**Mean±SD**	0.05 ± 0.17
**Median (range)**	0(0– 0.92)

CDVA: corrected distance visual acuity (Snellen chart); UDVA: uncorrected distance visual acuity (Snellen chart); SD: standard deviation

**Table 2:** Postoperative changes in refractive errors

**Variable**	**Preoperative**	**Postoperative**	**Change**	**Highest**	**Lowest**
**Objective refraction**					
**Sphere**				-1.75	-0.65
Mean ± SD	-1.28 ± 2.13	0.07± 0.71	-1.35±1.93		
Median (range)	-1.5 (-6.5 – 6)	0 (-1.5 – 2.5)	-1.5 (-6.75 – -6.25)		
**Cylinder**				-1.88	-1.45
Mean ± SD	-2.51 ± 1.45	-0.84 ± 0.94	-1.67 ± 1.03		
Median (range)	-2 (-7 – 1.25)	-0.5 (-5.25 – 0)	-1.5 (-4 – 1.75)		
**Spherical equivalent**				-2.60	-1.77
Mean ± SD	-2.54 ± 2.1	-0.35 ± 0.7	-2.18 ± 2.01		
Median (range)	-2.5 (-7.63 – 5.38)	-0.31 (-2.13 – 1)	-2.13 (-8.63 – 6)		
**Subjective refraction** *					
**Sphere**				-1.64	-0.79
Mean ± SD	-1.26 ± 2.14	-0.05 ± 0.52	-1.21 ± 2.05		
Median (range)	-1.5 (-6.5 – 6)	0 (-1.25 – 2.5)	-1.38 (-6.25 – 6.5)		
**Cylinder**				-2.26	-1.74
Mean ± SD	-2.46 ± 1.52	-0.46 ± 0.97	-2 ± 1.25		
Median (range)	-2 (3 – -7)	0 (-5 – 0)	-1.75 (-5.25 – 3.5)		
**Spherical equivalent**				-2.65	-1.78
Mean ± SD	-2.49 ± 2.13	0.28 ± 0.5	-2.22 ± 2.11		
Median (range)	-2.5 (-7.63 – 5.38)	0 (-2.5 – 0.63)	-2.38 (-7.63 – 6.25)		

* Subjective refraction deemed acceptable by patient; SD: standard deviation; P < 0.001 for all variables.

## Discussion

In this cross-sectional hospital-based study, we found that customized ablation PRK significantly reduced the degree of astigmatism. The postoperative change based on the results of an automated refraction exam was -1.67 ± 1.03 D (P < 0.001), and the postoperative change in subjective refraction was -2.00 ± 1.25 D (P < 0.001). For both PRK and laser-assisted in situ keratomileusis (LASIK), customized ablation has been shown to be more effective than the conventional approach (9, 15-18). For example, McDonald et al. showed that customized ablation PRK and LASIK led to low rates of higher-order aberrations in comparison to the conventional approach (13). Our findings concur with this result, as we found that, based on assessments at 6 months after surgery, customized ablation PRK was effective for treating astigmatism. The limitations of this study were its cross-sectional nature, relatively short follow-up period, and small sample size, which means that the presence of statistically significant changes does not imply an excellent outcome. Regardless of these limitations, the study was able to show that customized ablation PRK led to a significant improvement in astigmatism at 6 months after surgery. A long-term longitudinal study of the subjects in this study may help to refine our understanding of the effectiveness of customized ablation PRK for the treatment of astigmatism.
